# Update and Review on the Surgical Management of Primary Cutaneous Melanoma

**DOI:** 10.3390/healthcare2020234

**Published:** 2014-06-10

**Authors:** Solmaz Niknam Leilabadi, Amie Chen, Stacy Tsai, Vinaya Soundararajan, Howard Silberman, Alex K. Wong

**Affiliations:** 1 Division of Plastic and Reconstructive Surgery, Keck School of Medicine, University of Southern California, 1510 San Pablo St., Suite 415, Los Angeles, CA 90015, USA; E-Mails: solmazniknamMD@gmail.com (S.N.L.); amie.chen1202@gmail.com (A.C.); TsaiS1@mail.amc.edu (S.T.); vrljs92@yahoo.com (V.S.); 2 Department of Surgery, Keck School of Medicine, University of Southern California, 1510 San Pablo St., Suite 412, Los Angeles, CA 90015, USA; E-Mail: silberma@usc.edu

**Keywords:** primary cutaneous melanoma, melanoma, surgical management of melanoma, surgical margins of melanoma, sentinel lymph node biopsy in melanoma, lymphadenectomy in melanoma

## Abstract

The surgical management of malignant melanoma historically called for wide excision of skin and subcutaneous tissue for any given lesion, but has evolved to be rationally-based on pathological staging. Breslow and Clark independently described level and thickness as determinant in prognosis and margin of excision. The American Joint Committee of Cancer (AJCC) in 1988 combined features from each of these histologic classifications, generating a new system, which is continuously updated and improved. The National Comprehensive Cancer Network (NCCN) has also combined several large randomized prospective trials to generate current guidelines for melanoma excision as well. In this article, we reviewed: (1) Breslow and Clark classifications, AJCC and NCCN guidelines, the World Health Organization’s 1988 study, and the Intergroup Melanoma Surgical Trial; (2) Experimental use of Mohs surgery for *in situ* melanoma; and (3) Surgical margins and utility and indications for sentinel lymph node biopsy (SLNB) and lymphadenectomy. Current guidelines for the surgical management of a primary melanoma of the skin is based on Breslow microstaging and call for cutaneous margins of resection of 0.5 cm for MIS, 1.0 cm for melanomas ≤1.0 mm thick, 1–2 cm for melanoma thickness of 1.01–2 mm, 2 cm margins for melanoma thickness of 2.01–4 mm, and 2 cm margins for melanomas >4 mm thick. Although the role of SLNB, CLND, and TLND continue to be studied, current recommendations include SLNB for Stage IB (includes T1b lesions ≤1.0 with the adverse features of ulceration or ≥1 mitoses/mm^2^) and Stage II melanomas. CLND is recommended when sentinel nodes contain metastatic deposits.

## 1. Introduction

Melanoma accounts for 75% of all deaths related to skin cancer. It is a neoplasm in which there is the malignant transformation of melanocytes, the cells that are capable of forming the pigment melanin. Melanocytes arise most commonly in the skin of any part of the body, but may rarely occur in the eye and mucous membranes of various organs. The goal of this review is to discuss the surgical management of cutaneous melanoma.

## 2. Brief History, Staging Overview, and Current Guidelines

**Figure 1:**
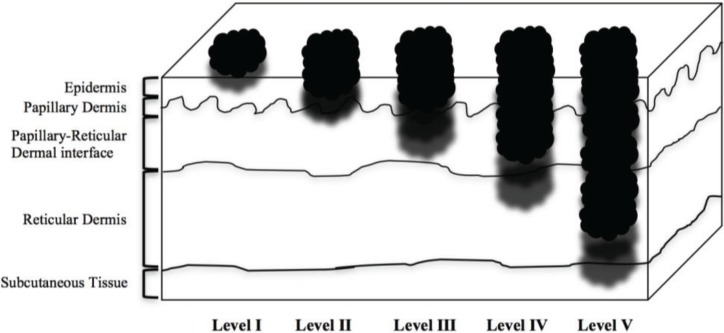
Layers of tissue and Clark level invasion.

Melanoma was first clinically described in 1820 by English general practitioner William Norris, who presented the first genetic, clinical, and epidemiologic features of the disease and also proposed treatment options, including the need for wide excision of skin and subcutaneous tissue to minimize recurrence [1]. In 1907, British physician W. Samson Handley [2] published an analysis of satellite metastases in which he advocated surgical margins of 2.54 cm, leading to guidelines in the 1970s and 1980s of 5 cm cutaneous excision margins independent of tumor thickness [3]. In 1969 and 1970, physicians Wallace H. Clark, Jr. and Alexander Breslow independently described tumor invasiveness and thickness relative to prognosis. Melanoma microstaging (T-stage) using the Clark Classification (Table 1) was based on the anatomic level of local invasion (Figure 1), whereas the Breslow Classification was based on the vertical thickness of the invasion in millimeters. In accordance with the recommendations of the American Joint Commission on Cancer (AJCC) [4], T microstaging now is determined exclusively by the Breslow classification. In the past several decades, surgical resection of the primary lesion and a margin of the surrounding normal skin based on the Breslow classification has been well studied and has led to increasingly conservative margins [5]. The National Comprehensive Cancer Network (NCCN) combined several large randomized prospective trials, such as the Intergroup Melanoma Surgical Trial [6] and the World Health Organization’s 1988 study [7], along with consensus from experience to propose current guidelines for melanoma excision. These guidelines recommend 0.5 cm cutaneous margins of resection for melanoma *in situ* (MIS), though the NCCN notes that >0.5 cm margins may be needed for lentigo melanoma (LM), 1.0 cm cutaneous margins for thin lesions defined as ≤1.0 mm thick, 1.0–2.0 cm for intermediate-thickness lesions defined as 1.01–2 mm thick, 2.0 cm for intermediate-thickness lesions defined as 2.01–4 mm thick, and 2.0 cm for thick lesions defined as >4 mm (Table 2) [8]. These recommendations are occasionally modified in the resection of lesions where there are aesthetic and functional concerns, for example, melanomas of the eyelid or cheek.

**Table 1:** Clark classification for melanoma, level of invasion.

Level I	Involving only the epidermis, *in situ*
Level II	Invasion of papillary dermis, does NOT reach papillary-reticular dermal interface
Level III	Invasion through papillary dermis, does NOT penetrate reticular dermis
Level IV	Invasion into reticular dermis
Level V	Invasion into subcutaneous tissue

**Table 2:** The National Comprehensive Cancer Network recommendations for surgical margins in melanoma excision.

Tumor microstage	Thickness	Margin
Melanoma *in situ* (Tis)		0.5 cm
Thin (T1)	≤1.0 mm	1.0 cm
Intermediate (T2)	1.01–2 mm	1.0–2.0 cm
Intermediate (T3)	2.01–4 mm	2.0 cm
Thick (T4)	>4 mm	2.0 cm

Under AJCC guidelines, the anatomic and prognostic staging group (Stages 0–III) is based on three characteristics: the tumor (T) microstage (Breslow level), the lymph node (N) classification (the pathologic status of the lymph nodes within the primary nodal basin associated with the anatomic location of the melanoma), and the metastasis (M) classification (the absence or presence, including the anatomic location, of distant metastatic disease) (Table 3, Table 4, Table 5 and Table 6). In general, the Breslow thickness correlates with prognosis: the thicker a tumor the greater chance that the melanoma will have spread. Within each T grouping, the prognosis is impaired by the finding of ulceration or one or more mitoses per square millimeter (Table 3, Table 4, Table 5 and Table 6). While thickness, ulceration, and mitoses have been used to define TNM categories and stage groupings, mitotic rate has now been identified as an independent predictor of survival. A multifactorial analysis of 10,233 patients identified it as the second most significant predictor of survival after thickness [9]. Thus, mitotic rate has been added as a required element in staging of melanoma [9].

**Table 3:** American Joint Committee on Cancer Tumor (T) Classification.

TX	Tumor cannot be assessed
T0	No evidence of tumor
Tis	Tumor remains on epidermis
T1a	Tumor ≤1.0 mm thick, without ulceration and mitosis <1/mm^2^
T1b	Tumor ≤1.0 mm thick, with ulceration or mitoses ≥1/mm^2^
T2a	Tumor is between 1.01 and 2.0 mm, without ulceration
T2b	Tumor is between 1.01 and 2.0 mm, with ulceration
T3a	Tumor is between 2.01 and 4.0 mm, without ulceration
T3b	Tumor is between 2.01 and 4.0 mm, with ulceration
T4a	Tumor >4.0 mm, without ulceration
T4b	Tumor >4.0 mm, with ulceration

**Table 4:** American Joint Committee on Cancer Regional Lymph Node (N) Classification.

NX	Lymph nodes cannot be assessed
N0	No spread to lymph nodes
N1a	Microscopic spread to 1 lymph node
N1b	Macroscopic spread to 1 lymph node
N2a	Microscopic spread to 2–3 lymph nodes
N2b	Macroscopic spread to 2–3 lymph nodes
N2c	In transit met(s)/satellite(s) *without* metastatic nodes
N3	4 or more metastatic nodes, or matted nodes, or in transit met(s)/satellite(s) *with* metastatic node(s)

**Table 5:** American Joint Committee on Cancer Distant Metastasis (M) Classification.

M0	No metastasis
M1a	Metastasis to distant skin, subcutaneous tissue, or distant lymph nodes
M1b	Metastasis to lungs
M1c	Metastasis to all other visceral sites, or any distant metastasis combined with elevated blood LDH level

**Table 6:** America Joint Committee on Cancer Anatomic Staging/Prognostic Groups.

Stage 0	Tis, N0, M0
Stage IA	T1a, N0, M0
Stage IB	T1b or T2a, N0, M0
Stage IIA	T2b or T3a, N0, M0
Stage IIB	T3b or T4a, N0, M0
Stage IIC	T4b, N0, M0
Stage IIIA	T1-T4a, N1a or N2a, M0
Stage IIIB	T1-T4b, N1a or N2a, M0 T1-T4a, N1b or N2b, M0 T1-T4a, N2c, M0
Stage IIIC	T1-T4b, N1b or N2b, M0 T1-T4b, N2c, M0 Any T, N3, M0
Stage IV	Any T, any N, any M

## 3. Surgical Diagnosis of Melanoma: Types of Biopsy

Pigmented lesions that are larger than several millimeters in diameter, are enlarging, variegated in pigmentation, and have irregular borders, or are ulcerated and/or bleeding raise the clinical suspicion of melanoma, and thus biopsy is indicated. The purpose of the biopsy is to establish the diagnosis of melanoma and, if positive, provide sufficient material to determine the indication for sentinel node analysis (discussed below) and establish the extent of the cutaneous margins that should be obtained at the time of definitive surgical excision. Although various biopsy techniques are described, including incisional, excisional, shave and punch biopsies, excisional biopsy is the technique that is highly recommended by the authors and both the NCCN and AJCC. It is the only technique that clearly establishes the diagnosis and definitive T classification [4,8,9]. Excisional biopsy eliminates the potential sampling errors of the partial excisions obtained with punch biopsy and incisional biopsy. The recommendations for an excisional biopsy are 1–3 mm and 1–2 mm margins from the NCCN [8] and the AJCC, respectively [4,9]. Partial incisional or punch biopsies are acceptable for larger lesions and should be taken from the most suspicious areas of the lesion. Shave biopsies are frequently problematical for surgical planning since they often transect the deep portion of the tumor, in which case the T classification is not definitively determined. Hence, the AJCC recommends against shave biopsies [4,9]. Although excisional biopsies are recommended, there are circumstances in which they may not be appropriate, including lesions with low suspicion for melanoma, large lesions with a diameter >2 cm, large facial or acral lesions, or where excision may be associated with aesthetic concerns [10,11].

## 4. Surgical Margins for Melanoma *in-Situ* (MIS)

While recent studies confirmed NCCN guidelines for thick and intermediate-thickness melanomas, data supporting optimal margins for MIS have not been as conclusive. Current NCCN guidelines (Version 2.2014) for MIS call for a 0.5 cm cutaneous margin of resection [12]. Greater margins may be required for large *in situ* lentigo maligna lesions. In recent years there has been considerable interest in applying Mohs micrographic surgery (MMS), useful in non-melanoma skin cancers [13], to the management of melanoma *in situ* in order to limit removal of non-diseased tissue in cosmetically sensitive areas [14]. MMS is a tissue-sparing surgery that allows for histological clearance with minimal amount of tissue excision. MMS for the treatment of melanoma has been controversial and experimental since the success of MMS depends on a contiguous tumor growth pattern, while melanoma is known to have satellite metastases [15]. However, there is mounting evidence that MMS for the treatment of melanoma could lead to equivalent or lower local recurrences and better survival rates compared to conventional surgery. One of the first studies to explore MMS *versus* wide margin excision in melanoma was done by Zitelli *et al.* in 1997 [16]. In this study, 535 consecutive patients with melanomas that varied in location and thickness underwent MMS and were followed for 5 years. The study revealed that when comparing each thickness group, 5-year survival and metastasis rates were equal or better in the MMS treated patients than the 15,798 historical control patients who underwent wide margin excision. Other such studies by Zitelli and colleagues that included more than 1000 cases over 15 and 22-year-periods have been extensively reviewed in several studies, including recent review papers by Whalen *et al.* [13] and Hui *et al.* [15] The Zitelli studies found a local recurrence rate of 0.5% for MIS when treated with MMS compared to 6% for MIS treated with standard surgery [17].

With increased utilization of MMS to accurately assess peripheral clearance, the adequacy of the standard 5 mm margins for melanoma *in situ* has been debated. In a large study by Kunishige *et al.* [18] of 1120 cases of MIS treated with MMS, the effectiveness of 9 mm compared to 6 mm surgical margins was confirmed with excision success rates of 86% for 6 mm margins and up to 98.9% for 9 mm margins; margin widths less than 6 mm were not studied. In addition, subtypes of MIS were not specified so it is not known whether the relatively larger margins could be due to a high percentage of cases with lentigo melanoma (LM), a subtype of MIS that is a more aggressive, invasive, and irregularly-shaped type of MIS in chronically sun-exposed areas. Despite the limitations, the study suggested that MIS could be treated as early invasive melanoma with surgical margins at least 9 mm. Erickson *et al.* [19] further discussed surgical margins for MIS, especially in regard to LM, by reviewing 15 studies from 1997 to 2008 that used MMS to assess clearance and minimize surgical margins. They found a reported recurrence rate of 6%–20% of LM associated with surgical margins of 5 mm, and thus concluded that margins larger than 5 mm may be required for the clinically ill-defined LM lesions. Although these studies call into question the adequacy of surgical margin of 0.5 cm for MIS, the NCCN recommendations continues to be 0.5 cm [8].

## 5. Surgical Margins for Thin Melanomas

The NCCN and AJCC recommendations for thin melanomas defined as ≤1.0 mm is excision with margins of 1.0 cm [4,8]. Although the latest AJCC guidelines now requires mitotic rate as an element to define the staging of the melanoma, the surgical margin of 1.0 cm remains for thin melanomas.

## 6. Surgical Margins for Intermediate-Thickness Melanomas

Data for melanomas of intermediate thicknesses have been well investigated in multiple large randomized trials. These trials include the WHO 1988 study [7] of 612 patients that compared 1 cm *vs.* 3 cm surgical margins for melanoma less than 2 mm-thick, the Intergroup trial [6] in 2000 that compared 2 cm *vs.* 4 cm surgical margins for 740 patients with 1–4 mm-thick melanomas, and the Thomas *et al.* [20] trial in 2004 of 900 patients that compared 1 cm *vs.* 3 cm margins for melanomas thicker than 2 mm. Results from the WHO and Intergroup study suggested that intermediate-thickness melanomas could be treated by 1–2 cm excision margins. The Thomas trial found that 1 cm margins were associated with higher local recurrence for melanomas thicker than 2 mm compared to 3 cm margins, though with similar overall survival rates [6,7,20]. These findings generated the NCCN guidelines for intermediate-thickness melanomas and further divided them into the two categories of 1.01–2 mm and 2.01–4 mm with recommended clinical margins of 1.0–2.0 cm and 2.0 cm, respectively [8]. In a multicenter randomized trial, Gillgren *et al.* studied 936 patients from 1992 to 2004 with melanomas thicker than 2 mm and compared 2 cm *vs.* 4 cm surgical excision margins and found no difference in the 5-year overall survival (65%) between the two groups [21]. When patients were stratified into groups based on Breslow classification of tumor thickness, the Gillgren trial confirmed the safety of 2 cm margins for melanoma in the intermediate-thickness category (2.01–4 mm).

## 7. Surgical Margins for Thick Melanomas

Relative to the abundance of data for the intermediate-thickness melanomas, there is a paucity of data for thick melanomas. For thick melanomas (>4 mm), it is unclear whether margins exceeding 2 cm affect local recurrence and survival rates owing to the fact that thick melanomas may reflect biologic aggressiveness of the melanoma that cannot be cured through wide excision alone [5]. A 1998 study by Heaton *et al.* found that excisional margins wider than 2 cm had no significant benefits to local control or disease free survival [22]. However, a recent prospective study in 2013 of 637 patients conducted at the Melanoma Institute Australia found that patients that underwent 2 cm or less surgical margins for thick melanomas (>4 mm) were found to have twice the risk of local recurrence compared with those who had >2 cm surgical margins [23]. The studies by Thomas *et al.* [20] and Gillgren *et al.* [21] described above in the intermediate-thickness melanomas section were limited to melanomas thicker than 2 mm; however, they did not differentiate between 2.01–4 mm intermediate and >4 mm thick melanomas. The current standard according to NCCN and AJCC is excision margins of 2 cm for thick (>4 mm) melanomas [4,8].

## 8. Management of Regional Lymph Nodes: Sentinel Lymph Node Biopsy and Lymphadenectomy

### 8.1. Introduction

An important prognostic indicator for melanoma is the disease status of regional lymph nodes inasmuch as the initial melanoma metastasis is likely to occur through the lymphatic system. The first one or several regional lymph nodes to receive metastatic deposits are designated the “sentinel lymph nodes”. The pathology of melanoma suggests that sentinel lymph node (SLN) involvement may then be followed by spread of the cancer to more distal regional nodes [24]. In the absence of clinically detectable nodes, SLN biopsy (SLNB) has now been adopted as the standard of care, depending on the T-stage (see below). To locate the SLNs (which are clinically occult) at surgery, two tracers are available. Lymphoscintigraphy using a radioisotope, Tc-99, injected around the tumor within 24 hours of surgery, results in radioactivity in the SLNs, which is detected intraoperatively with a gamma probe. The second tracer is a vital dye (usually lymphazurin blue or methylene blue) that is injected intradermally at the periphery of the lesion at the time of surgery. The vital dye travels to the SLNs and stains them blue. Surgeons may use one or both markers to determine the nodes to be removed. The use of both markers is associated with a slightly higher identification rate of the SLNs. If the SLNs are free of metastatic disease it is highly unlikely that the remaining regional nodes bear metastases (see below). SLN biopsy has prognostic value and can determine the stage of melanoma without subjecting a patient to the heretofore recommended elective lymph node dissection (ELND) [25].

When compared to a standard complete lymphadenectomy, a SLNB is less invasive and has minimal complication risks [24]. If the SLNB is positive for metastases by light microscopy or immunohistochemistry, a completion lymph node dissection (CLND), where the rest of the lymph nodes are explored and examined, is currently recommended [12,26,27,28]. Prior to CLND systemic staging by CT or CT-Pet scan should be considered [12]. CLND serves two purposes: the first is to more accurately assess the stage and prognosis of the melanoma and the second is to prevent relapse in the nodal basin. As noted above, if a SLNB is negative for metastatic disease, it is most likely that the other non-sentinel nodes in the basin are negative for cancer cells as well [29]. However, distant disease can occasionally occur without nodal metastases, but positive regional nodes are more likely the major markers for distant disease [25]. Patients with clinically detectable lymph nodes proven by preoperative biopsy to be positive for metastases, undergo a therapeutic lymph node dissection (TLND) and forego a SLNB [30]. CLND when the SLNB is positive is currently the recommended standard care for melanoma in accordance with guidelines jointly promulgated by the Society of Surgical Oncology and the American Society of Clinical Oncology [26,27].

### 8.2. Utility of Sentinel Lymph Node Biopsy

Thin melanomas less than 1.0 mm of Breslow thickness have a very low rate of positive SLN, <5%, and therefore SLNB is not indicated and may be inappropriate for these patients [31,32,33]. Coit *et al.* [34] further discussed that it is uncommon for melanomas of 0.75 mm or thinner to have risk factors such as lymphovascular invasion, ulceration, and high mitotic rate. Unless these risk factors are present, a wide excision alone is warranted, otherwise NCCN guidelines indicate a SLN biopsy is warranted [34]. Mozzillo *et al.* [35] conducted a retrospective analysis of patients with thin melanomas ≤1.0 mm and found that the rate of positive SLN was 4.9%. Mitotic rate was the only clinicopathologic factor associated with SLN positivity, suggesting that SLNB should be standard care for patients with such tumors [35]. Previous long-term follow-up studies have found that a small but definite percentage of patients with thin melanomas developed regional nodal recurrence and distant metastases [36,37]. For such tumors, the most significant predictor of SLN positivity was mitotic rate and Breslow depth [36,37,38,39]. The current NCCN recommendations [8] do not recommend SLNB for patients with MIS or melanoma ≤1.0 mm without adverse features (1 or more mitoses per square mm or ulceration). They do recommend discussions with the patient about SLNB for melanomas ≤1.0 mm with these adverse features and for melanomas >1.0 mm.

A false-negative result from a SLNB ranges from 1.5% to 4.1%, except in the case of head and neck melanomas where the false-negative rate ranges from 3.3% to as high as 44% [40,41,42,43,44]. However, it is widely accepted as an important staging tool for melanomas and a strong independent predictor of outcomes in melanomas 1.2–3.5 mm in thickness [45]. Histopathological thickness of the melanoma (*i.e.*, Breslow microstaging) has been the most reliable predictor of SLN status. Tumors of intermediate thickness have been found to benefit most from the SLNB staging. SLNB for intermediate thickness melanomas is most useful in identifying patients who have early micro-metastases and who then have an indication under current guidelines for regional lymphadenectomy as well as adjuvant therapy [31,32,46]. Even so, there is some variability as to what is considered an intermediate thickness tumor based on Breslow thickness. Landi *et al.* [31] studied 455 melanoma patients and found that intermediate thickness melanomas of 0.76 to 4.0 mm obtain the most prognostic value from SLNB. Lens *et al.* [46] reviewed 12 studies through 2001 for a total of 4218 patients and concluded that Breslow thickness correlates with the presence of sentinel node metastasis. Incidence of positive SLNB increased as thickness of the melanoma increased. Melanomas between 1.51 and 4.0 mm were found to have a significantly higher risk of a positive SLNB compared to melanomas between 1.0 and 1.5 mm thickness. Therefore, SLNB serves as an effective and appropriate staging procedure for melanomas between 1.51 and 4.0 mm. Mays *et al.* [32] noted that although melanomas between 1.0 to 2.0 mm thick are likely to have a negative SLNB, patients with these melanomas exhibit diversity in the biologic behavior of their tumor; however, it has been difficult to indicate another prognostic factor to identify a subset of these patients who would benefit from SLNB. Therefore, SLNB is recommended for all patients with a tumor between 1.01 to 2.0 mm [4,32].

SLNB for thick melanomas >4 mm in Breslow thickness has been questioned due to the poor prognosis and to the high risk of hematogenous dissemination in this subset of patients [47,48,49]. Oliveira Filho *et al.* [48] found that regardless of the histopathological SLN status of thick melanomas, patients had similar rates of recurrence and mortality. According to such findings, SLNB may not be indicated for patients with thick melanomas [48]. In contrast, SLN status has been found to be the most important prognostic factor for survival and may serve as a useful tool to stratify patients in this high-risk group into adjuvant trials [31,50]. Furthermore, some physicians have found that a negative SLN status may identify a subset of patients with a more favorable prognosis and who may be long-term survivors [46,47]. For example, Cherpelis *et al.* [47] found that SLN status was predictive of disease-free survival for patients with thick melanomas. Consensus is that SLNB is currently indicated in all thick melanomas with no evidence of systemic metastasis on CT or PET imaging.

In summary, current AJCC guidelines call for SLNB for patients with T1b or T2 tumors (*i.e.*, clinical stages IB and II) [4].

### 8.3. Utility of Completion Lymph Node Dissection

Following a positive SLN result, patients are offered CLND as part of the standard care protocol as discussed above. Of concern, however, is that a CLND may result in surgical morbidity. Previous reports have noted that only 14% to 28% of patients with positive SLNs harbor disease in other regional lymph nodes [51,52,53]. Similar evidence of a low incidence of non-sentinel node involvement was observed in the *First Multicenter Selective Lymphadenectomy Trial* (MSLT I) [28]. These data indicate that CLND would be an unnecessary procedure for that large subset of patients with positive SLNs but no non-sentinel node involvement. Thus, CLND would be of potential benefit if the patients likely to harbor metastatic disease in non-SLN could be identified [54]. In various analyses, an array of features appear to correlate with non-sentinel node involvement, including ulceration, satellitosis, neurotropism, multiple positive SLNs, a SLN with multifocal involvement or tumor deposits >2 mm, extranodal extension, capsular involvement, and primary tumor thickness >2 mm [55]. However, the absence of these features is not sufficiently predictive of disease-free non-sentinel nodes to alter the current standard of care when the SLN is positive.

Whether or not CLND provides a survival advantage for patients who have undergone the procedure remains a topic of debate. Previous studies have demonstrated a survival advantage for melanoma patients who underwent CLND compared to melanoma patients who underwent therapeutic lymph node dissection (TLND) after developing clinically palpable disease. A German multicenter retrospective study found a 12% survival benefit at 5 years for patients who had a positive SLNB result followed by CLND compared to patients who underwent a later TLND [56]. A study by Morton *et al.* found a 5 year survival advantage of 22% for patients who underwent immediate lymphadenectomy after positive SLN biopsy results as opposed to delayed lymph node dissection (DLND) for nodal disease that developed during a period of clinical observation [57]. Pasquali *et al.* [58] conducted a retrospective series and reported a difference of 18% in the 5 year overall survival rate between patients in the CLND group compared to patients in the TLND group. The percentage of survival benefit reported was similar to the value reported in other studies, however it lacked statistical significance. In contrast, Rutkowski *et al.* conducted a retrospective analysis and found no significant difference in overall survival and disease-free survival between the group of patients that underwent CLND after a positive SLNB result *versus* the group of patients who underwent TLND due to clinical lymph node metastasis [59]. Recently, in February 2014, the New England Journal of Medicine published the Final Trial Report of Sentinel-Node Biopsy *versus* Nodal Observation in Melanoma which compared 2 groups: (1) underwent wide excision and SLN biopsy with CLND if the SLN was positive for disease; and (2) underwent wide excision alone with observation alone and a TLND if a node becomes clinically evident. This study found that the 10-year disease free survival for group 1 *versus* group 2 in melanomas of intermediate thickness 1.20–3.50 mm was 71.3% *vs.* 64.7% (*p* = 0.01) respectively and in thick melanomas >3.50 mm, 50.7% *vs.* 40.5% (*p* = 0.03) respectively [60]. Furthermore, the 10-year melanoma specific survival for melanomas ≥1.20 mm was 62.1% *vs.* 41.5% in the two groups respectively [60]. This shows that a CLND improves disease free and melanoma free survival when a SLN biopsy is positive compared to observation alone.

Pilko *et al.* [61] compared the overall survival of patients with a melanoma of Clark level III and a Breslow thickness of 1.00 mm or more or melanomas of Clark level IV or V and any Breslow thickness. Patients were sorted into three groups: (1) patients with a positive SLNB who then underwent CLND; (2) patients initially clinically node negative who presented with clinically positive nodes during observation who then underwent DLND; and (3) patients who had synchronous primary melanoma and regional lymph node metastases. Patients with synchronous primary melanoma and regional lymph node metastases had the poorest 5 year overall survival. Patients who had a CLND for a SLN metastasis with a tumor diameter <5.0 mm had significantly better overall survival compared to patients who had DLND. Patients with a positive SLN with a tumor diameter >5.0 mm had similar survival as patients with synchronous primary melanoma and regional lymph node metastasis [61]. These results emphasize the prognostic heterogeneity among stage III melanoma patients, particularly among patients with positive SLNs.

### 8.4. Arguments against Sentinel Lymph Node Biopsy and Completion Lymph Node Dissection

SLNB and CLND are invasive procedures that may be associated with various postoperative complications. The MSLT I trial reported an acute complication rate of 10% in patients who had SLNB alone and 37% in patients who had CLND [62]. The Sunbelt Melanoma Trial saw a 4.6% total complication rate among patients who underwent SLNB alone and a 23.2% rate in patients who had both SLNB and CLND. The risk for lymphedema was 0.66% for patients who had SLNB alone and 11.7% for patients with CLND [63].

It has been cited that only 20% of patients will have a positive SLNB result following the procedure and that of those patients only 20% will have non-SLNs that are positive for metastatic disease [64]. Therefore, 96% of patients will have undergone a SLNB and CLND that proved unnecessary in retrospect [65].

However, with the current results from the MSLT-I [60], it has shown that the advantage of SLN biopsy and CLND is clear in 10-year disease free survival and melanoma specific survival.

## 9. Conclusions

Current guidelines for the surgical management of a primary melanoma of the skin are based on Breslow microstaging and call for cutaneous margins of resection of 0.5 cm for MIS, 1.0 cm for melanomas ≤1.0 mm thick, 1–2 cm for melanoma thickness of 1.01–2 mm, 2 cm margins for melanoma thickness of 2.01–4 mm, and 2 cm margins for melanomas >4 mm thick.

Although, the role of SLNB, CLND, and TLND continue to be studied, current recommendations include SLNB for Stage IB (includes T1b lesions ≤1.0 with the adverse features of ulceration or ≥1 mitoses/mm^2^) and Stage II melanomas. CLND is recommended when sentinel nodes contain metastatic deposits.
